# The Influence of Individual Set-Pieces in Elite Rink Hockey Match Outcomes

**DOI:** 10.3390/ijerph182312368

**Published:** 2021-11-24

**Authors:** Jordi Arboix-Alió, Guillem Trabal, Raúl Hileno, Joan Aguilera-Castells, Azahara Fort-Vanmeerhaeghe, Bernat Buscà

**Affiliations:** 1 Department of Sports Science, Ramon Llull University, FPCEE Blanquerna, 08025 Barcelona, Spain; jordiaa1@blanquerna.url.edu (J.A.-A.); joanac1@blanquerna.url.edu (J.A.-C.); azaharafv@blanquerna.url.edu (A.F.-V.); bernatbs@blanquerna.url.edu (B.B.); 2 Department of Physical Activity Sciences, University of Vic–Central University of Catalonia, 08500 Vic, Spain; 3 National Institute of Physical Education of Catalonia (INEFC), University of Lleida, 25192 Lleida, Spain; rhileno@gencat.cat

**Keywords:** performance analysis, roller hockey, match variables, binary logistic regression, explanatory modeling

## Abstract

The main objective of this study was to analyze the influence of individual set-pieces (Free Direct Hits and Penalties) in elite rink hockey match outcomes in different game situations. A sample of 161 matches played between high-standard teams during ten consecutive seasons (2009–2010 to 2018–2019) were analyzed using a binary logistic regression. The full evaluated model was composed of an explanatory variable (set-pieces scored) and five potential confounding and interaction variables (match location, match level, match importance, extra time, and balanced score). However, the final model only included one significant interaction variable (balanced score). The results showed that scoring more individual set-pieces than the opponent was associated with victory (OR = 6.1; 95% CI: 3.7, 10.0) and was more relevant in unbalanced matches (OR = 19.5; 95% CI: 8.6, 44.3) than in balanced matches (OR = 2.3; 95% CI: 1.2, 4.5). These findings indicate that individual set-pieces are strongly associated with match outcomes in matches played between high-standard teams. Therefore, it is important for teams to excel in this aspect, and it is suggested that these data can encourage coaches to reinforce the systematic practice of individual set-pieces in their training programs. Additionally, it is suggested that teams have specialist players in this kind of action to mainly participate in these specific match moments.

## 1. Introduction

Rink hockey, also known as roller hockey or hardball hockey, is a team sport played by two teams of five players on a rectangular rink (40 m × 20 m) surrounded by a one-meter high barrier. Like other sports, the increasing social interest and economic impact of team sports have resulted in several studies about the influence of the match variables on the final outcome [1,2,3,4]. In recent years, research focused on rink hockey match variables and other performance indicators has risen considerably [5,6,7]. In this vein, match analysis appears to be widely accepted by players, coaches and sports scientists as an important source of information to analyze and subsequently improve sports performance [8,9]. Moreover, it is especially helpful in providing objective reference knowledge about the strengths and weaknesses of opponents [10]. Additionally, match analysis contributes to developing the players’ technical and tactical knowledge, critical thinking, decision-making and confidence [11,12]. Therefore, it seems necessary to identify the most relevant performance indicators in every sport.

Previous rink hockey research has reported the influence of different match variables. Home advantage has been one of the most studied topics, thus determining its effect at around 60% [7,13], similar to that of other team sports [14]. Other studies have analyzed match variables such as scoring the first goal [6], scoring sequence [15] and the influence of opponents’ offensive play on the goalkeepers’ performance [16].

Among these different rink hockey match variables, individual set-pieces such as free direct hit (FDH) or penalty (PEN) are probably the most relevant aspects influencing the match outcome. Indeed, it is reported that 21.08% of goals scored in the First Spanish Division (*OkLiga*) are achieved from individual set-pieces (11.58% by PEN and 9.49% by FDH) [17]. These set-pieces are particular events involving a direct opposition between the FDH or PEN shooter and the goalkeeper. In FDH, the shooter has five seconds to start the execution (from 7.4 m), being able to choose a direct shot or approaching and dribbling towards the goalkeeper to score, while in the PEN, the shooter has five seconds to start the execution, consisting of a direct shot on the goal from the penalty point (5.4 m).

Formerly, the PEN and FDH happened only occasionally during a match. However, a new rink hockey regulation aiming to increase the number of goals per match went into effect in the 2009–2010 season. The main purpose of this new regulation was to encourage a more offensive style and, consequently, more strictly penalize the fouls committed. These new rules, such as the 45-s ball possession limit per attack, the accumulation of ten team fouls, and the subsequent penalization with an FDH or the temporary numerical inferiority when a player is sanctioned with a blue card were factors that generated more scoring chances (5.93 goals per match before and 7.13 goals after the 2009–2010 regulation) [13]. Among the aforementioned modifications, perhaps the most important was the change regarding offences that have incurred FDH and PEN since the total number of set-pieces per match increased.

There is a popular belief among coaches, players and rink hockey enthusiasts that individual set-pieces are one of the most determining factors in matches played between high-standard teams and that, often, the match result can be decided by the success in these actions. However, to the best of our knowledge, no research has been found to analyze this. Given the gap in the scientific literature, further research is warranted to establish the influence of individual set-pieces. Therefore, the main objective of this study was to analyze the influence of individual set-pieces in rink hockey match outcomes in different game situations. Given the paucity of available data assessing the association between set-piece success and the final match outcome, a true hypothesis was challenging to generate. However, following popular belief, it was hypothesized that set-piece performance would be associated with match outcomes in rink hockey matches.

## 2. Materials and Methods

### 2.1. Sample

A total of 161 matches during ten consecutive seasons (2009–2010 to 2018–2019) were analyzed. All games were chosen from the following competitions: World Cup, European Cup, WS Europe Cup (CERS Cup), Champions League, Continental Cup, Intercontinental Cup, Spanish Copa del Rey, Portugal Cup, Italian Cup, Supercopa de España, Supercoppa Italiana and Supertaça de Portugal. In order to avoid a difference-level bias, only matches between high-standard teams were included. Therefore, the analyzed matches were only the semi-finals (*n* = 82) and finals (*n* = 79) of the aforementioned competitions. In each game, the data from both teams were recorded separately. Only matches in which there were set-piece goals were included.

### 2.2. Design and Procedures

Data were collected by professional technicians of the league. To assess data reliability, 100 individual set-pieces were selected, and two different observations were performed to assess intra- and inter-rater reliability. The consensus surpassed 90% on all criteria and categories (intra-observer *κ* = 0.992; inter-observer *κ* = 0.984). In addition, a generalizability analysis was carried out [18] using SAGT software, version 1.0 (Málaga, Spain) [19] (Table 1). Following suggestions from Blanco-Villaseñor et al. [20], two measurements were made for assessment: (a) the results of generalizability (number of individual set-pieces that made up the sampling) and (b) the observation instrument’s validity; (a) the generalizability coefficient (relative and absolute = 0.996) corresponding to the measurement plan [Categories]/[set-pieces] establishes that, with the number of set-pieces analyzed, high reliability of generalization precision is obtained; (b) regarding the measurement, plan [set-pieces]/[Categories], the generalizability coefficient (relative and absolute = 0.000) guarantees, in the theoretical framework of the Theory of Generalizability, the validity of the designed observation instrument [20,21].

Match outcome was used as the outcome variable. The number of individual set-pieces scored was used as the explanatory variable. Finally, five covariates were examined using binary logistic regression to identify their possible confounder or modifier effect on the relationship between the set-pieces scored and match outcome (Table 2).

### 2.3. Statistical Analysis

A binary logistic regression model was built for explanatory purposes. This model made it possible to measure the adjusted effect of the explanatory variable (X) on a response (Y) in the presence of possible confounding or interaction variables (X_p_) that could confuse or modify the effect of X on Y.

In the first step of the statistical modeling process, a *full model* was specified consisting of an explanatory variable (SPSco), five confounding variables (MatLoc, MatLev, MatImp, ExtTim, BalSco) and five interaction terms (SPSco × MatLoc, SPSco × MatLev, SPSco × MatImp, SPSco × ExtTim, SPSco × BalSco). Considering the number of events observed in the sample (144 matches won) and the *one in ten rule* (i.e., one parameter can be studied for every ten events) proposed by Peduzzi et al. [22], at most, 14 parameters could be included in this initial model:

Logit(MatOut) = α + β × SPSco + γ_1_ × MatLoc + γ_2_ × MatLev + γ_3_ × MatImp + γ_4_ × ExtTim + γ_5_ × BalSco + δ_1_ × SPSco × MatLoc + δ_2_ × SPSco × MatLev + δ_3_ × SPSco × MatImp + δ_4_ × SPSco × ExtTim + δ_5_ × SPSco × BalSco

In the second step, the significance of the set of first-order interactions was evaluated using a global likelihood ratio test (*chunk test*). If the result of this test was not statistically significant (*p* > 0.05), all interactions were eliminated from the model. In contrast, if the result was statistically significant (*p* ≤ 0.05), individual likelihood ratio tests were applied to each interaction separately, and only those that were statistically significant were retained in the model.

The third step evaluated whether the confounding variables that did not belong to significant interactions should remain in the model as adjustment variables. For each value of the significant moderator variables, the odds ratio of the study factor (OR_SPSco_) was estimated in the *reference model* (model containing all confounding terms and only significant interaction terms) and all possible reduced models (submodels derived from the reference model in which one or more confounding terms were eliminated). Next, we determined whether the OR_SPSco_ estimated in the reduced models represented a change of more than 10% with respect to the OR_SPSco_ estimated in the reference model. If the change was greater than 10% (practically important change), the evaluated submodel was rejected. On the other hand, if the change was less than or equal to 10% (practically not important change), the submodel was preselected and its precision assessed to examine whether its confidence intervals (OR_SPSco_ 95% CI) were narrower than those of the reference model.

Once the modeling process was completed and the *final model* was selected, the absence of collinearity (variance inflation factor (VIF)) and over-dispersion (residual mean deviance (RMD)) was verified in the main estimated logistic regression models (full, reference, final and simple). The linearity assumption was not tested because all the variables were categorical.

Finally, the effect of individual set-piece performance (SPSco) on the final match outcome (MatOut) was estimated both in the final model (adjusted OR) and the simple model (crude OR). In addition, due to the difficult interpretation of the OR as a measure of association, a rough estimate of the proportion ratio (PR) with their respective 95% CIs was made from the marginal estimates of the probabilities of each event.

Statistical analyses were done using Stata/IC v.16.1 statistical package (Stata Corporation, College Station, TX, USA). The confounding assessment was evaluated with the postestimation commands estimation store and lrtest. The confusion was assessed with the user-written command confound; OR calculation was performed with the postestimation command lincom, and the approximate estimation of the PR was made with the user-written command adjrr.

## 3. Results

Table 3 shows the descriptive and inferential analysis of the individual set-pieces studied. The confidence interval for a proportion (1-α confidence interval for π) was calculated using the Wilson method.

The result of the *chunk test* indicated the statistical significance of the set of first-order interactions of the full model ($\chi_{LR}^{2}$ = 18.54, *df* = 6, *p* = 0.005). Only one significant interaction was detected from the individual likelihood ratio tests: SPSco × BalSco ($\chi_{LR}^{2}$ = 15.23, *df* = 1, *p* = 0.0001). Consequently, the SPSco × MatLoc ($\chi_{LR}^{2}$ = 1.13, *df* = 2, *p* = 0.569), SPSco × MatLev ($\chi_{LR}^{2}$ = 0.0004, *df* = 1, *p* = 0.983), SPSco × ImpMat ($\chi_{LR}^{2}$ = 0.44, *df* = 1, *p* = 0.507), and SPSco × ExtTim ($\chi_{LR}^{2}$ = 1.32, *df* = 1, *p* = 0.251) interactions were removed from the full model and did not become part of the reference model.

Fifteen reduced models were built from the reference model. In these models, three terms were set by the hierarchical principle (SPSco, BalSco, SPSco × BalSco), and between one and four terms were excluded to assess their possible confounding effect (MatLoc, MatLev, MatImp, and ExtTim). In all the ORs estimated in the reduced models (OR_SPSco|BalSco__=__Unbalanced_ y OR_SPSco|BalSco__=__Balanced_), changes less than 10% were detected with respect to the ORs estimated in the reference model. Consequently, all reduced models became finalists. However, only the model that included the adjustment variable BalSco and the SPSco × BalSco interaction was selected as the final model to estimate the effect of SPSco on MatOut because it was the most parsimonious final model and had a higher precision than the reference model.

Table 4 shows the *b* coefficients and the OR (e^*b*^) of the main estimated models during the modeling process as well as the likelihood, global significance and diagnoses, highlighting the fulfilment of the collinearity assumptions (mean VIF ≤ 10) and equidispersion (RMD ≈ 1).

**Figure 1:**
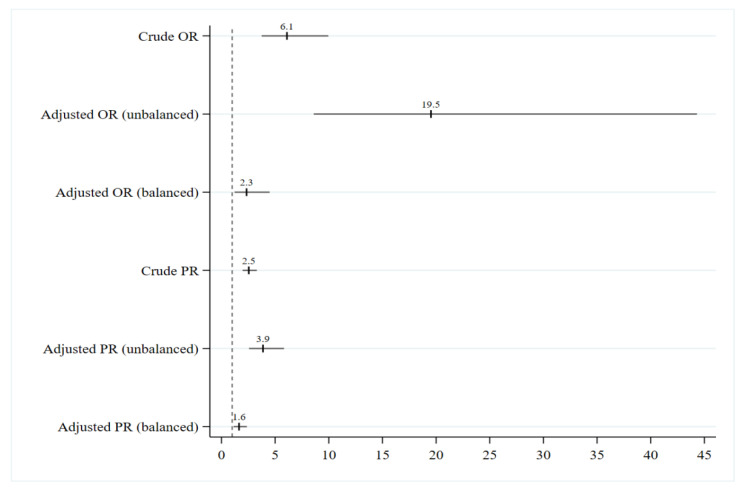
Effect of scoring more individual set pieces than the opponent on the matches won odds/proportion.

Figure 1 shows the effect of SPSco on MatOut as a function of BalSco. This figure indicates that, in unbalanced matches, scoring more individual set-pieces than the rival team multiplied the odds of winning the match by OR_SPSco|BalSco = Unbalanced_ = e^2.97−2.12×0^ = 19.5 (95% CI: 8.6 a 44.3). Otherwise, in balanced matches, scoring more set-pieces than the rival team multiplied the odds of winning the match by OR_SPSco|BalSco = Balanced_ = e^2.97−2.12×1^ = 2.3 (95% CI: 1.2 a 4.5).

Taking the PR values into account, in unbalanced matches, scoring more individual set-pieces than the rival multiplied the proportion of matches won by PR_SPSco|BalSco = Unbalanced_ = 3.9 (95% CI: 2.6 a 5.8). Otherwise, in balanced matches, scoring more set-piece goals than the rival team multiplied the proportion of matches won by PR_SPSco|BalSco = Balanced_ = 1.6 (95% CI: 1.1 a 2.4).

## 4. Discussion

The main purpose of this study was to determine how the set-piece performance affects the match outcome in different game situations. The main finding was that scoring more individual set-pieces than the opponent was associated with victory in rink hockey (OR = 6.1; 95% CI: 3.7, 10.0) and was more relevant in unbalanced matches (OR = 19.5; 95% CI: 8.6, 44.3) than in balanced matches (OR = 2.3; 95% CI: 1.2, 4.5). To the best of the authors’ knowledge, this is the first study to demonstrate the popular belief among rink hockey followers and coaches that the success of individual set-pieces is a determining factor in match outcome. Despite the lack of studies available to compare the present results, these findings are in line with Arboix-Alió, Trabal, Aguilera-Castells, et al. [17], who reported higher set-piece effectiveness in the best-ranked teams at the end of the Spanish league season. In the aforementioned study, teams classified in the *Euroleague* group (1st to 4th position) achieved more FDH goals than the other teams. Moreover, the same authors reported significantly different goalkeeper performances depending on the final ranking of their teams. The results showed that goalkeepers of the *Euroleague* group teams saved more PEN and FDH (72.81%) than the *Permanency* (9th to 12th position) or *Relegation* (13th to 16th position) groups’ teams (65.22% and 61.77%, respectively).

### 4.1. Balanced Score

Interestingly, scoring more individual set-pieces than the opponent became more relevant in unbalanced matches. This strong association can be explained by the internal logic of rink hockey (e.g., rally dynamics, number of players, court dimensions) and the kind of matches analyzed in the present study (similar standard teams). During the unbalanced-score case scenario between two different-standard teams, the losing team usually adopts a conservative strategy to avoid a blowout. However, in matches played between high-standard teams, when one team achieves an advantage of two or more goals, the match becomes frenetic. This causes the losing team to pressure the opponent to get back into the match, forcing both teams to play in a box-to-box style. In this particular scenario, the losing team uses a riskier style, pressing its opponents across the entire court. This creates situations in which it is easier to commit defensive fouls or receive blue cards, which are sanctioned with an FDH. Indeed, the appropriate contextual situation in which the winners increase their set-piece effectiveness is created in these kinds of matches. In a recent study, Arboix-Alió et al. [23] reported that players were more successful with a favourable score than when they were losing or drawing. Thus, the probability of scoring a penalty when winning by three goals was 3.83 times higher than when drawing. Moreover, the odds of scoring a free direct hit when winning by two goals was 2.40 times higher than when drawing.

### 4.2. Match Location

Match location had no significant interaction in the effect of scoring more set-pieces than the opposing team on the odds of won matches. Surprisingly, it seems that hockey players do not benefit from individual set-pieces in the home advantage (HA) effect, reported to be around 60% in rink hockey [7,13]. The present results agree with previous investigations, which reported no influence of match location in either rink hockey [5,23] or ice hockey [24]. According to Casimiro [25], this lack of home advantage effect could be explained by the fact that individual set-pieces are specific events between the shooter and the goalkeeper and are less influenced by certain variables that explain home advantage, such as court dimensions, type of surface or game plan [26,27].

Moreover, it is important to note that most of the analyzed matches were played in a neutral court, while the rest consisted of two-stage knock-out competition. Despite this not being evident in rink hockey, previous investigations reported a lower second-leg home advantage effect compared with the regular season in football [28,29].

### 4.3. Match Level

Match level had no significant interaction in the effect of scoring more set-pieces than the opposing team on the odds of won matches. One feasible reason for this lack of level effect could be that only high-standard teams were analyzed. In fact, the same analyzed teams played against each other (e.g., F.C Oporto vs. S.L Benfica or F.C Barcelona vs. Liceo H.C) in both national (e.g., Portuguese or Spanish Cup) and international competitions (e.g., Champions League).

### 4.4. Match Importance

The kind of match (semi-final or final) had no significant interaction in the effect of scoring more set-pieces than the opposing team on the odds of won matches. This lack of significance could be attributed to the same reasons that explain the lack of interaction of match level. In rink hockey competitions with a level bias [30,31], the same teams competed for the championship across the different seasons.

Despite the usefulness of these findings, the present investigation also has some limitations that should be acknowledged and addressed in future studies. Firstly, the present study has not considered the possible effects of other factors such as refereeing, travel or spectators’ behaviour. In future investigations, it would be interesting to consider these aspects and the match moment where set-pieces happen. On the other hand, further research should replicate our findings in other rink hockey competitive contexts, such as the female hockey league or lower levels of competition (grassroots sport or minor leagues). In addition, the technical actions of players have not been analyzed. Finally, the lack of studies of rink hockey reduces the possibility of identifying some tendencies between findings. The strengths of our study lie in its novelty, as this is the first study to analyze the set-piece influence in rink hockey match outcome and the number of analyzed set-pieces during a ten-year period of the most prestigious rink hockey competitions.

## 5. Conclusions and Practical Applications

In conclusion, the current study reported that individual set-pieces were strongly associated with match outcomes in rink hockey, indicating that when teams scored more individual set-pieces than their opponents, the odds of winning the match multiplied by 6.1. This kind of action became especially relevant in matches where there was a difference in the score of higher than two goals at some point in the match.

In light of these results, rink hockey coaches should be encouraged to increase the systematic practice of FDH and PEN shots in their training programs. The design of individual set-pieces practice needs to be innovative to recreate the levels of anxiety, distraction and perceptions of control raised by such high-pressure situations. Moreover, scouting the goalkeeper and set-piece shooters of opposing teams seems essential for success. Additionally, these findings suggest the necessity of teams having specialist players in this kind of action. Considering the rink hockey regulation that allows the unlimited substitution of players during any match moment, it could be decisive for a team to have a specialist goalkeeper for FDH or a PEN shooter who only participates in these specific match moments.

## Figures and Tables

**Table 1:** Results corresponding to the generalizability design [Categories] [Set-pieces].

	SC	*df*	Mean Square	Random	Mixt	Corrected	%	Standard Error
**[set-pieces]**	0.33	636	0.001	−0.005	−0.005	−0.005	0	0
**[cat]**	1014.638	26	39.025	0.061	0.061	0.061	30.645	0.016
**[set-pieces][cat]**	2284.621	16536	0.138	0.138	0.138	0.138	69.355	0.002

**Table 2:** Description of the analyzed variables.

Role	Variable	Categories	Description
Outcome	Match outcome (MatOut)	Not won (0)	The analyzed team lost or tied the match
		Won (1)	The analyzed team won the match
Explanatory	Set-pieces scored (SPSco)	Less or equal (0)	The analyzed team scored equal or fewer goals from set-pieces than the rival
		More (1)	The analyzed team scored more goals from set-pieces than the rival
Covariate	Match location (MatLoc)	Away (1)	The analyzed team played away from home
		Neutral (2)	The analyzed team played on neutral ground
		Home (3)	The analyzed team played at home
	Match level (MatLev)	National (0)	The analyzed match was from a national competition
		International (1)	The analyzed match was from an international competition
	Match importance (MatImp)	Semifinal (0)	The analyzed match was a semifinal
		Final (1)	The analyzed match was a final
	Extra time (ExtTim)	No (0)	Extra time was not reached in the analyzed match
		Yes (1)	Extra time was reached in the analyzed match
	Balanced score (BalSco)	Unbalanced (0)	At some point in the match, there was a difference in the score higher than 2 goals
		Balanced (1)	At no point in the match was there a difference in the score higher than 2 goals

Note. Within each variable, the category with the lowest numerical code (e.g., the category Not won in MatOut variable) was considered as the reference category in the constructed logistic regression model.

**Table 3:** Descriptive and inferential analysis of the categorical variables used to build the binary logistic regression model.

Variable	Categories	*n*	%	95% CI of π	
				LL	UL
Match outcome (MatOut)	Not won (0)	178	55.3	49.8	60.6
	Won (1)	144	44.7	39.4	50.2
Set-pieces scored (SPSco)	Less or equal (0)	190	59.0	53.6	64.2
	More (1)	132	41.0	35.8	46.4
Match location (MatLoc)	Away (1)	63	19.6	15.6	24.2
	Neutral (2)	196	60.9	55.4	66.0
	Home (3)	63	19.6	15.6	24.2
Match level (MatLev)	National (0)	176	54.7	49.2	60.0
	International (1)	146	45.3	40.0	50.8
Match importance (MatImp)	Semifinal (0)	164	50.9	45.5	56.3
	Final (1)	158	49.1	43.7	54.5
Extra time (ExtTim)	No (01)	254	78.9	74.1	83.0
	Yes (1)	68	21.1	17.0	25.9
Balanced score (BalSco)	Unbalanced (0)	158	49.1	43.7	54.5
	Balanced (1)	164	50.9	45.5	56.3

Note. *n* = number of observations; CI = confidence interval; π = population proportion converted to percentage; LL = lower limit; UL = upper limit.

**Table 4:** Main estimated logistic regression models.

	Full Model		Reference Model		Final Model		Simple Model	
Variables	*B*	OR	*b*	OR	*b*	OR	*b*	OR
SPSco	2.55 **	12.81 **	2.99 ***	19.81 ***	2.97 ***	19.52 ***	1.81 ***	6.10 ***
	[0.80, 4.30]	[2.24, 73.37]	[2.16, 3.82]	[8.63, 45.44]	[2.15, 3.79]	[8.60, 44.31]	[1.32, 2.30]	[3.74, 9.96]
MatLoc2	0.05	1.05	0.34	1.41				
	[−0.81, 0.91]	[0.45, 2.49]	[−0.36, 1.04]	[0.70, 2.83]				
MatLoc3	−0.15	0.86	0.21	1.24				
	[−1.21, 0.91]	[0.30, 2.49]	[−0.61, 1.03]	[0.54, 2.81]				
MatLev	0.20	1.22	0.21	1.23				
	[−0.54, 0.94]	[0.58, 2.56]	[−0.36, 0.77]	[0.70, 2.17]				
MatImp	0.27	1.31	0.13	1.13				
	[−0.41, 0.95]	[0.67, 2.58]	[−0.39, 0.65]	[0.67, 1.91]				
ExtTim	−1.47 **	0.23 **	−1.01 **	0.36 **				
	[−2.51, −0.43]	[0.08, 0.65]	[−1.71, −0.31]	[0.18, 0.73]				
BalSco	1.01 **	2.75 **	0.87 *	2.40 *	0.52	1.69		
	[0.28, 1.75]	[1.32, 5.73]	[0.17, 1.58]	[1.19, 4.83]	[−0.13, 1.18]	[0.88, 3.25]		
SPSco × MatLoc2	0.72	2.06						
	[−0.72, 2.17]	[0.49, 8.73]						
SPSco × MatLoc3	0.81	2.25						
	[−0.90, 2.52]	[0.41, 12.39]						
SPSco × MatLev	−0.01	0.99						
	[−1.18, 1.16]	[0.31, 3.19]						
SPSco × MatImp	−0.36	0.70						
	[−1.43, 0.71]	[0.24, 2.03]						
SPSco × ExtTim	0.85	2.35						
	[−0.62, 2.33]	[0.54, 10.23]						
SPSco × BalSco	−2.33 ***	0.10 ***	−2.05 ***	0.13 ***	−2.12 ***	0.12 ***		
	[−3.52, −1.14]	[0.03, 0.32]	[−3.12, −0.99]	[0.04, 0.37]	[−3.17, −1.07]	[0.04, 0.34]		
Constant	−1.52 **	0.22 **	−1.68 ***	0.19 ***	−1.28 ***	0.28 ***	−0.98 ***	0.38 ***
	[−2.61, −0.43]	[0.07, 0.65]	[−2.59, −0.76]	[0.07, 0.47]	[−1.78, −0.77]	[0.17, 0.46]	[−1.29, −0.66]	[0.27, 0.52]
LL	−176.6		−178.4		−183.1		−192.5	
LR chi-squared	89.61 ***		86.06 ***		76.63 ***		57.83 ***	
Mean VIF	3.68		1.71		2.08		1.00	
RMD	1.15		1.14		1.15		1.20	

Note. confidence intervals in brackets. *b* = regression coefficient; OR = odds ratio; LL = log likelihood; LR = likelihood ratio; VIF = variance inflation factor; RMD = residual mean deviance. * *p* < 0.05, ** *p* < 0.01, *** *p* < 0.001.
